# Representation of Older Adults in COVID-Related Newspaper Coverage

**DOI:** 10.1093/geroni/igab046.3727

**Published:** 2021-12-17

**Authors:** Mijin Jeong, Sarah Jen, Hyun Kang, Michael Riquino, Jaime Goldberg

**Affiliations:** 1 University of Kansas, LAWRENCE, Kansas, United States; 2 University of Kansas, Lawrence, Kansas, United States; 3 University of Wisconsin-Madison, Madison, Wisconsin, United States

## Abstract

Based on clinical and epidemiological evidence, COVID-19 infection can occur in people of all ages; however, the media typically focuses its attention on the vulnerability of older adults and individuals with chronic illnesses. This study aims to explore the representation of older adults during the first month of the pandemic in the U.S. by comparing the narratives of older adults and younger adults in national media sources. A systematic search identified 115 articles published in four major newspapers in the U.S. included USA Today, The New York Times, Los Angeles Times, and The Washington Post between March 11 and April 10, 2020 in which older adults and younger adults were quoted on topics related to the intersection of COVID-19 and aging. Quotes were inductively reviewed using thematic content analysis. In 115 articles, there were 265 quotes from older adults (n=104, 39%) and younger adults (n=161, 61%). When comparing patterns that were common or distinctive between older and younger individuals quoted, three key themes emerged: 1) impacts of COVID-19 on older adults and resulting vulnerability, 2) debated perspectives over the value of older adults’ lives, and 3) a counternarrative of resiliency among older adults. This study provides the opportunity to understand how the pandemic may impact representations of older adults and findings emphasize the importance of voice among older adults to combat ageist messaging and promote counternarratives to assumptions of vulnerability. Also, it suggests for policymakers and practitioners to insight into how the representation of older adults is disseminated by media.

